# Change in activity patterns in the prefrontal cortex in different phases during the dual-task walking in older adults

**DOI:** 10.1186/s12984-023-01211-x

**Published:** 2023-07-07

**Authors:** Chang Yoon Baek, Hyeong Dong Kim, Dong Yup Yoo, Kyoung Yee Kang, Jang Woo Lee

**Affiliations:** 1 Department of Physical Therapy and School of Health and Environmental Science, College of Health Science, Korea University, Seoul, South Korea; 2 Department of Rehabilitation Medicine, National Health Insurance Ilsan Hospital, 100 Ilsan-Ro, Ilsandong-Gu, Goyang-Si, Gyeonggi-do 10444 South Korea

**Keywords:** Prefrontal cortex, Dual-task walking, Aging, Older adult

## Abstract

**Background:**

Studies using functional near-infrared spectroscopy (fNIRS) have shown that dual-task walking leads to greater prefrontal cortex (PFC) activation compared to the single-task walking task. However, evidence on age-related changes in PFC activity patterns is inconsistent. Therefore, this study aimed to explore the changes in the activation patterns of PFC subregions in different activation phases (early and late phases) during both single-task and dual-task walking in both older and younger adults.

**Methods:**

Overall, 20 older and 15 younger adults performed a walking task with and without a cognitive task. The activity of the PFC subregions in different phases (early and late phases) and task performance (gait and cognitive task) were evaluated using fNIRS and a gait analyzer.

**Results:**

The gait (slower speed and lower cadence) and cognitive performance (lower total response, correct response and accuracy rate, and higher error rate) of older adults was poorer during the dual task than that of younger adults. Right dorsolateral PFC activity in the early period in older adults was higher than that in younger adults, which declined precipitously during the late period. Conversely, the activity level of the right orbitofrontal cortex in the dual-task for older adults was lower than for younger adults.

**Conclusions:**

These altered PFC subregion-specific activation patterns in older adults would indicate a decline in dual-task performance with aging.

## Introduction

Gait is a semiautomatic function, which requires attention for locomotor control [1, 2]. Attentional demand is lower during normal walking, and higher for difficult ones such as dual-task walking [1, 3]. Studies using functional near-infrared spectroscopy (fNIRS), which enables real-time monitoring of brain activation during dual-task walking to confirm the association between prefrontal cortex (PFC) activity and dual-task walking, have reported that dual-task walking leads to greater PFC activation than that with single-task walking [2, 4]. Hence, dual-task walking is related to executive function, i.e., the PFC is responsible for higher-order cognitive processes [5, 6]. However, the cognitive and motor deficits caused by aging necessitate activation of greater areas in the brain or overactivation of the same brain area in older adults to ensure performance similar to that of younger adults (i.e., compensatory mechanisms) [7, 8]. A decline in gait performance and increased PFC activation are observed during walking and simultaneously subtracting the number 3 sequentially in older adults compared to younger adults [9]. Ohsugi et al. reported higher PFC activation in older adults during the dual task compared to younger adults [10]. However, Holtzer et al. reported that PFC activity was greater in younger adults compared to older adults [11]. Therefore, evidence from previous studies is inconsistent. Several studies have suggested that such discrepancies can arise due to methodological differences, including the nature of the secondary tasks employed and suboptimal number and positioning of channels [9, 12–14]. Previous studies have assessed only one PFC subregion during dual-task walking [e.g., the dorsolateral PFC (DLPFC) or rostral PFC (RPFC)] using fNIRS with a limited number of channels [9, 13, 15–17]. The DLPFC, ventrolateral PFC (VLPFC), RPFC, and orbitofrontal cortex (OFC) are the PFC subregions involved in dual-task performance and adaptation to unpredictable circumstances [18–21]. Since fNIRS with a limited number of channels is not well-suited for visualizing the network and the function of subregions during dual-task walking [22], it is recommended to utilize a type of fNIRS that can measure all PFC subregions [20, 22, 23]. Hence, we hypothesized that assessing these PFC subregions would help identify definite differences in PFC activity during dual tasks between older and younger adults. Furthermore, while fNIRS has the advantage of providing information about the timing and dynamics of neural responses during dual-task walking compared to other neuroimaging techniques [12], many studies that included older and younger adults have addressed task-related activity in the PFC in only one phase of the task [11, 15, 17, 19]. Exploring the activity patterns in different phases (early and late phases) while measuring all PFC subregions may better elucidate the difference in PFC activation patterns between older and younger adults [24, 25]. One study demonstrated that PFC activation in younger adults was higher in the early and late periods during the dual task than that during the single task [25]. In another study, the level of PFC activity in older adults was higher in the early period than that in the late period during the walking task [24]. A study by Holtzer et al. revealed that in older adults, the activity levels of the PFC consistently remained higher during consecutive dual-task walking trials compared to the single-task walking condition [26] Unlike the studies mentioned above that solely measured PFC activity during tasks without considering intervals, this study focuses on investigating changes in activation in different subregions of the PFC and their activation patterns during intervals. This approach would yield invaluable insights into age-related alterations in brain activation during different phases and performance. We aimed to investigate the difference in dual-task performance between older and younger adults and the difference in activation patterns in the PFC subregions in different phases (early and late phases) between older and younger adults. We hypothesized that dual-task performance of older adults would be worse than that of younger adults, and that older adults would exhibit excessive PFC activation in the early period and a steep decline in the late period compared to younger adults during dual-task performance, based on the evidence that age-related decline in brain resources leads to poor functional brain activity (i.e., over-recruitment and reduced “ceiling” of recruitment) [27].

## Materials and methods

### Participants

This study was approved by the Institutional Review Board of the National Health Insurance Service Ilsan Hospital and conducted in accordance with the principles of the Declaration of Helsinki.This study enrolled 35 right-handed participants, of which 20 were older adults (mean age, 67.05 ± 1.82 years; years of education, 14.10 ± 1.77) and 15 were younger adults (mean age, 28.47 ± 3.65 years; years of education, 15.00 ± 1.00). All tests were conducted in public hospitals from September 20, 2021, to October 27, 2021. All participants provided written informed consent before inclusion in the study. The inclusion criteria were as follows: (1) older adults aged between 65 and 80 years and younger adults aged between 20 and 40 years; and (2) Korean-Montreal Cognitive Assessment (K-MoCA) score greater than 23, which indicates normal global cognitive function [28]. The exclusion criteria were as follows: (1) any neurological disorders such as stroke and Parkinson’s disease, or orthopedic disorders that could potentially affect gait ability; and (2) subjects who use a device while walking.

### Procedure

Participants were instructed to walk back and forth for 40 s on a 20 m walkway equipped with a gait analyzer (5 s) in the center, while wearing an fNIRS device in each condition (single- and dual-task conditions). PFC activation and gait pattern were analyzed simultaneously in each condition. In the dual-task condition, participants performed a cognitive task (serial subtraction of sevens) while walking. The single condition did not include the cognitive task. All participants were asked to maintain a static standing position for 40 s before commencing the walking task, with the following instruction: “stand as comfortably as possible without thinking”. Thereafter, each participant walked for 40 s after receiving the instruction to “start”. In the case of the dual-task condition (walking + serial subtraction of sevens), participants were given a three-digit number between 100 and 200 along with the instruction “start” by the tester. The subtraction task was designated as a secondary task because it provoked higher PFC activity during dual-task walking and provided consistency of PFC activation in functional magnetic resonance imaging (fMRI) studies [14, 29]. All participants had intact global cognitive function, according to the K-MoCA. Hence, we used serial subtraction of sevens as the secondary task, which entails greater attentional demand than serial subtraction of threes [30]. Additionally, many studies with PFC activation and task performance used subtraction of sevens to determine dual-task interference in older and younger adults [13, 14, 16, 31]. Furthermore, all participants received the following instruction to prevent task prioritization in the dual-task condition: “walk such that your attention is distributed evenly between walking and subtraction.” All trials were performed twice and the values were averaged accordingly.

### fNIRS measurement

**Fig. 1:**
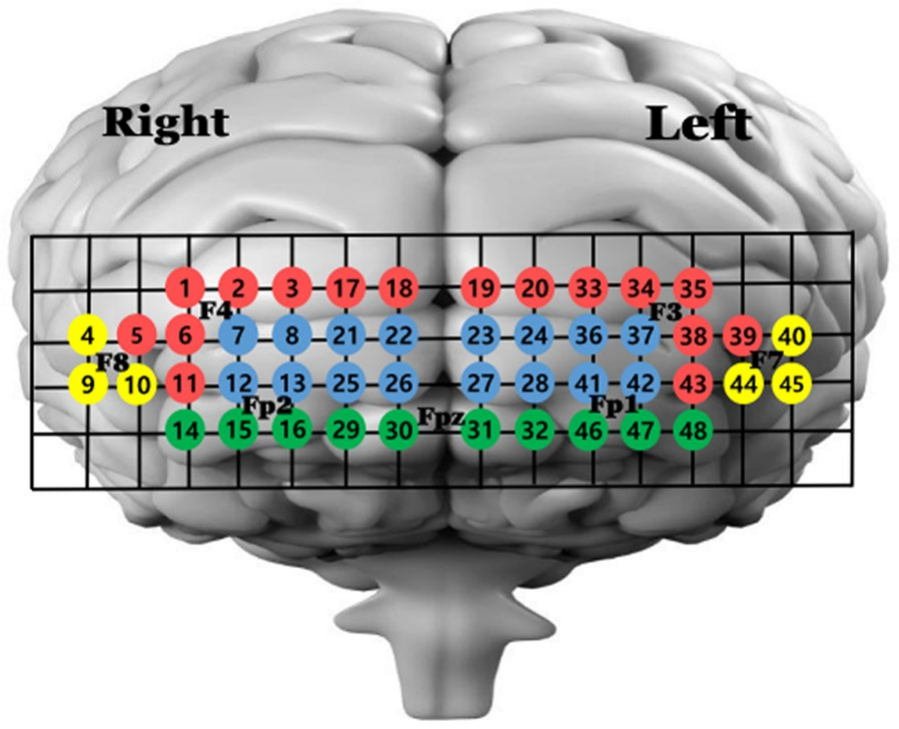
Mapping of prefrontal cortex subregions using 48 channels. The numbers represent channels. Red color channels: dorsolateral prefrontal cortex, blue color channels: rostral prefrontal cortex, yellow color channels: ventrolateral prefrontal cortex, green color channels: orbitofrontal cortex

We used a 48-channel fNIRS (NIRSIT; OBELAB Inc., Seoul, Korea) device for optimal measurement of the activity patterns in the PFC subregions, which means that it covered the requisite PFC subregions [23], unlike short-channel fNIRS that can measure only one PFC subregion [17, 32]. PFC activity was expressed as the oxygenated hemoglobin (HbO) value, which is more reliable for measuring gait-related changes in PFC activity than deoxygenated hemoglobin [33]. This instrument consists of 24 lasers and 32 detectors with wavelengths of 780 nm and 850 nm at a 8.138-Hz sampling rate. The differential pathlength factor (DPF) that corresponded to the wavelengths was fixed at 6 and 5.2. Therefore, the source-detector distance was set to 3 cm to account for spatial resolution and DPF, which could obtain more signals from the cerebral layer than the extracerebral layers, indicating a depth of approximately 15‒20 mm from the scalp [34, 35]. The FPz was mapped in accordance with the international 10‒20 electroencephalography system, such that the participant’s forehead was covered with the device [2]. Before measurement of task-related activity in the PFC, the gain of the detector was calibrated individually for each participant to minimize the effects of different skin pigments, age-dependent skin and skull thickness, and other biological factors [20, 23, 35]. Based on previous research that used 48-channel fNIRS, mapping of the DLPFC (right side: 1, 2, 3, 5, 6, 11, 17, and 18; left side: 19, 20, 33, 34, 35, 38, 39, and 43 channels), RPFC (right side: 7, 8, 12, 13, 21, 22, 25, and 26; left side: 23, 24, 27, 28, 36, 37, 41, and 42), VLPFC (right side: 4, 9, and 10; left side: 40, 44, and 45 channels), and OFC (right side: 14, 15, 16, 29, and 30; left side: 31, 32, 46, 47, and 48 channels) was established (Fig. 1) [20, 23, 36]. The regions of interest (ROIs) in the PFC subregions were drawn according to the results of previous fNIRS studies [20, 36]. The measurement Sections (80 s) consists of a rest section of 40 s and task section of 40 s. Data processing was performed for 30 s, excluding the first and last 5 s in each section to eliminate anticipatory reactions. Furthermore, to measure the changes in the patterns during different phases, the task period (30 s) was divided into an early-activity period of 15 s and a late-activity period of 15 s. Hence, the value of the rest section was subtracted from that of each task section (early and late periods to determine the relative change in HbO) [9]. After the completion of one trial, the participant rested for more than 60 s to reduce the responsiveness of PFC activation. We obtained the average HbO value of each subregion [23]. All walking tasks were carried out in a tranquil, dimly lit room, with the presence of only two evaluators and one subject, in order to minimize the impact of external factors, including the surrounding environment. During and after the subject's evaluation, one evaluator conducted continuous visual inspection using the NIRSIT program.

### Cognitive and physical function measurement

Global cognitive function was assessed using the K-MoCA score. We used the Trail Making Test A (TMTA) and Trail Making Test B (TMTB) minus TMTA (TMTB-A), respectively, to measure cognitive processing speed and executive function [37]. The TMTA requires the participant to draw a line to connect 25 numbers in ascending order. The TMTB requires the participant to alternately connect numbers and letters (1-A-2 to L-13). Evaluation of cognitive performance (serial subtraction of sevens) in the single-task condition was performed with the participant in the sitting position for 40 s. The parameters of cognitive performance included total response, correct response, incorrect response, accuracy rate (correct response/total response × 100, %), and error rate (incorrect response/correct response × 100, %). Additionally, in the dual-task condition, the above-mentioned cognitive parameters were measured and recorded using a recorder while walking. Participants were given a 15-min break between cognitive task assessment in the single-task condition and gait-task assessment, including the single-task and dual-task conditions, to avoid the effect of learning on cognitive performance. A gait analyzer (OptoGait, Microgate Srl, Bolzano, Italy) measuring 5 m in length and 1.8 m in width was installed at the center of a 20-m walkway, which has excellent reliability and strong validity in overground tests in healthy and injured adults [38, 39]. When the participant passed through this section, the gait analyzer’s sensor bars, which are equipped with light-emitting diodes, detected the participant’s gait pattern, including speed, stride length, gait variability (SD stride time/mean stride time × 100, %), cadence, swing phase time, and double-limb support (DLS) phase time. As the participant walked, the gait parameters were recorded in the 5-m straight section using a sensor bar before the first turn [39, 40]. The order of single-task and dual-task walking was randomized. Additionally, the short physical performance battery (SPPB), which evaluated gait, sitting to standing, and balance abilities (score range: 0 to 12), was used to confirm mobility deficits in older adults [41]. We used the Geriatric Depression Scale (GDS) (score range: 0 to 30) to assess depression [42], and the Falls Efficacy Scale-International (FES-I) (score range: 14 to 64) to assess the fear of falls in older adults [43]. These measures were implemented to minimize the influence of any factors that might potentially affect the dual-task ability in older adults [44].

### Data processing

NIRSIT data analysis was performed using MATLAB (Version 2019b; MathWorks Inc., Natick, Mass., USA). All data were processed in accordance with the recommendations of a review of fNIRS as much as possible [2, 22]. The first measured raw signals were converted to the logarithmic change in optical density, and the optical density data were converted to HbO using the modified Beer-Lambert method. During the data processing, unreliable and poor signal data were excluded by establishing a signal-to-noise threshold of less than 20 dB. The number of removed channels did not exceed 10% of the total channels. A 4th order Butterworth low-pass and high-pass filtering at 0.1 and 0.01, respectively, was employed to minimize the effect of respiration (0.2 Hz), heart rate (0.5 Hz), Mayer’s waves (0.1 Hz), device noise, and other irrelevant physiological noise. Furthermore, a motion artifact removal filter was employed (Accel threshold, 1.8; Gyro threshold, 1.8; envelope window, 100; motion OD Xcorr, 0.6; contact window, 10). To mitigate the influence of head movements during walking and turning, we applied motion artifact reduction algorithm to process the signals [20]. The algorithm effectively reduced the presence of inappropriate signals. The turning interval in walking tasks was excluded from the data processing period when measuring task-related activity in the PFC. The analyst utilized the NIRSIT analysis program for ongoing monitoring to identify and label the turning sections, with the objective of eliminating the identified turning sections. During the data processing stage, channels that displayed sharp spikes or baseline movements, indicating sudden and significant deviations from the expected signal, as well as discontinuities primarily caused by motion artifacts such as facial movements, were manually excluded. Furthermore, outliers that exceeded 2.58 standard deviations from the mean were removed and replaced with a zero value. The signals with the desired pattern were manually selected via visual inspection to supplement the ROI [34, 45]. After the data processing, guided by an expert, a subsequent visual inspection of the data was conducted once again.

### Statistical analysis

All statistical analyses were performed using SPSS 18.0 (IBM SPSS, Chicago, IL, USA). All values were presented as means and standard deviations. Based on the G ∗ power 3.1 calculation, with an effect size of 0.25 and a power of 0.80, a sample size of 25 participants was needed. On the basis of previous findings that showed an interaction effect between time and task factors on PFC activity (F = 8.91, partial η^2^ = 0.21), 35 participants were needed [10]. The independent t-test and chi-squared test were used to compare the characteristics of the two groups. We used a 2 × 2 × 2 mixed-design analysis of variance (ANOVA), with age (younger vs. older) as the between-subjects variable and task (single- vs. dual-tasks) and time period (early vs. late period) as the within-subjects variables, to verify the effect of age, task, and time period on PFC activity. For the dual-task performance (gait and cognitive task) analysis, a 2 × 2 mixed-design ANOVA was performed, with age (younger vs. older adult) as the between-subjects variable and task (single- vs. dual- task) as the within-subjects variable. A post-hoc test with Bonferroni correction was used to interpret the results of the significant interaction effects. The significance level was set at P < 0.05. Furthermore, Pearson’s correlation analysis was used to determine the association between activity in the PFC subregions and dual-task performance.

## Results

### Characteristics of participants

The K-MoCA scores, education period, and gait speed did not differ significantly between the older and younger participants. However, the results of the TMTA and TMTB-A differed significantly between the two groups. Deficits in mobility, depression, and fear of falling were not observed in older adults according to the results of the SPPB (cutoff score: ≤ 9), GDS (cutoff score: ≥ 11), and FES-I (cutoff score: > 23), respectively (Table 1).

**Table 1:** characteristics of participants

Characteristic	Older adults (N = 20)	Younger adults (N = 15)	P
Age, years	67.05 ± 1.82	28.47 ± 3.65	< 0.001
Sex			
Male/female	14/6	8/7	0.313
MoCA score	26.95 ± 1.82	28.13 ± 0.99	0.074
Education, years	14.10 ± 1.77	15.00 ± 1.00	0.073
TMTA, s	44.49 ± 8.24	28.82 ± 5.97	< 0.001
TMTB-TMTA, s	73.16 ± 29.08	27.62 ± 7.86	< 0.001
Total response, n	15.06 ± 4.03	16.97 ± 3.77	0.163
Correct response, n	14.75 ± 4.12	16.60 ± 3.85	0.185
Incorrect response, n	0.31 ± 0.52	0.37 ± 0.60	0.751
Accuracy, %	97.76 ± 3.64	97.63 ± 3.43	0.915
Error, %	2.44 ± 4.06	2.55 ± 3.74	0.932
Gait speed, m/s	1.20 ± 0.11	1.23 ± 0.07	0.261
SPPB, score	11.10 ± 1.12	NA	NA
GDS, score	8.75 ± 1.89	NA	NA
FES-I, score	18.50 ± 3.90	NA	NA

A chi-square test was performed for sex, while independent t-tests were conducted for the remaining variables. FES-I, Falls Efficacy Scale-International; GDS, Geriatric Depression Scale; K-MoCA, Korean-Montreal Cognitive Assessment; SPPB, Short Physical Performance Battery; TMTA, Trail Making Test A; TMTB, Trail Making Test B

### Cognitive performance (serial subtraction of sevens)

The main effect of task was observed on the total response [*F* (1,33) = 168.873, P < 0.001, partial η^2^ = 0.837), correct response [*F* (1,33) = 145.171, P < 0.001, partial η^2^ = 0.815], accuracy rate [*F* (1,33) = 7.400, P < 0.05, partial η^2^ = 0.183], and error rate [*F* (1,33) = 7.691, P < 0.01, partial η^2^ = 0.189]. This result indicated that performing the cognitive task in the single-task condition provided a higher total correct response, correct response, and accuracy rate and lower error rate in serial subtraction of sevens compared to the dual-task condition. The interaction effect of task × group was observed on the total response [*F* (1,33) = 13.362, P < 0.01, partial η^2^ = 0.288], correct response [*F* (1,33) = 13.578, P < 0.01, partial η^2^ = 0.292], accuracy rate [*F* (1,33) = 4.749, P < 0.05, partial η^2^ = 0.126], and error rate [*F* (1,33) = 4.808, P < 0.05, partial η^2^ = 0.127], which means that older adults showed a lower total response, correct response and accuracy rate, and higher error rate during the dual-task compared to younger adults, but not during the single task (Table 2). The TMTA score was correlated with the total response, correct response, accuracy rate, and error rate during the dual-task condition. The TMTB-A score was correlated with the total response and correct response during the dual-task condition (Table 3).

**Table 2:** Comparison of performance

	Older adults (N = 20)		Younger adults (N = 15)		Main effect		Interaction effect
	Single task	Dual task	Single task	Dual task	Task	Group	Task by Group
Motor task							
Speed, m/s	1.20 ± 0.11	1.10 ± 0.11^a^	1.23 ± 0.07	1.21 ± 0.14^f^	*P* = 0.002 (*F* = 11.778)	*P* = 0.032 (*F* = 5.013)	*P* = 0.027 (*F* = 5.339)
Stride length, cm	126.70 ± 11.87	120 ± 10.67^a^	132.62 ± 10.70	129.53 ± 12.79^f^	*P* < 0.001 (*F* = 16.664)	*P* = 0.50 (*F* = 4.138)	*P* = 0.157 (*F* = 2.096)
Variability, %	1.90 ± 0.79	2.27 ± 0.93	1.47 ± 0.65	1.62 ± 0.64^f^	*P* = 0.145 (*F* = 2.226)	*P* = 0.011 (*F* = 7.299)	*P* = 0.523 (*F* = 0.416)
Cadence, step/min	117.73 ± 5.40	110.86 ± 5.51^a^	120.77 ± 7.51	117.82 ± 7.8^c, e^	*P* < 0.001 (*F* = 47.249)	*P* = 0.023 (*F* = 5.681)	*P* = 0.010 (*F* = 7.530)
Swing phase, s	0.37 ± 0.02	0.37 ± 0.02	0.38 ± 0.02	0.39 ± 0.04^f^	*P* = 0.309 (*F* = 1.067)	*P* = 0.060 (*F* = 3.805)	*P* = 0.112 (*F* = 2.667)
Double limb support phase, s	0.30 ± 0.04	0.33 ± 0.03^a^	0.30 ± 0.03	0.32 ± 0.05^c^	*P* < 0.001 (*F* = 17.494)	*P* = 0.540 (*F* = 0.384)	*P* = 0.364 (*F* = 0.848)
Cognitive task							
Total response, n	15.06 ± 4.03	7.89 ± 3.47^a^	16.97 ± 3.77	12.95 ± 4.60^a, e^	*P* < 0.001 (*F* = 168.873)	*P* = 0.010 (*F* = 7.402)	*P* = 0.001 (*F* = 13.362)
Correct response, n	14.75 ± 4.12	7.10 ± 3.63^a^	16.60 ± 3.85	12.53 ± 4.56^a, d^	*P* < 0.001 (*F* = 145.171)	*P* = 0.008 (*F* = 8.006)	*P* = 0.001 (*F* = 13.578)
Incorrect response, n	0.31 ± 0.52	0.79 ± 0.96^c^	0.37 ± 0.60	0.42 ± 0.82	*P* = 0.080 (*F* = 3.273)	*P* = 0.464 (*F* = 0.550)	*P* = 0.142 (*F* = 2.263)
Accuracy rate, %	97.76 ± 3.64	88.88 ± 13.58^b^	97.63 ± 3.43	96.65 ± 5.84^f^	*P* = 0.010 (*F* = 7.400)	*P* = 0.080 (*F* = 3.254)	*P* = 0.037 (*F* = 4.749)
Error rate, %	2.44 ± 4.06	13.81 ± 17.22^a^	2.55 ± 3.74	3.88 ± 7.33^f^	*P* = 0.009 (*F* = 7.691)	*P* = 0.071 (*F* = 3.468)	*P* = 0.035 (*F* = 4.808)

^a^p < 0.001, between single- and dual-task

^b^p < 0.01, between single- and dual-task

^c^p < 0.05, between single- and dual-task

^d^p < 0.001, between groups in dual-task condition

^e^p < 0.01, between groups in dual-task condition

^f^p < 0.05, between groups in dual-task condition

**Table 3:** Relationship between dual task performance and PFC activation and TMTA & B-A

Dual task condition	TMTA			TMTB-A		
	r	P-value	95% CI	r	P-value	95% CI
Gait speed	− 0.288	0.093	− 0.549, − 0.012	− 0.250	0.147	− 0.548, 0.084
Stride length,	− 0.493	0.003	− 0.695, − 0.225	− 0.427	0.011	− 0.696, − 0.083
Variability	0.284	0.076	− 0.319, 0.328	0.337	0.047	− 0.037, 0.620
Cadence	− 0.368	0.030	− 0.625, − 0.048	− 0.260	0.131	− 0.516, 0.059
Swing phase	− 0.154	0.377	− 0.439, 0.280	− 0.211	0.225	− 0.444, 0.115
Double limb support phase	0.134	0.442	− 0.199, 0.483	0.073	0.678	− 0.235, 0.399
Total response	− 0.656	< 0.001	− 0.808, − 0.489	− 0.548	< 0.001	− 0.696, − 0.391
Correct response	− 0.700	< 0.001	− 0.836, − 0.533	− 0.600	< 0.001	− 0.741, − 0.445
Incorrect response	0.346	0.042	0.064, 0.622	0.367	0.030	0.025, 0.669
Accuracy rate	− 0.529	0.001	− 0.725, − 0.294	− 0.504	0.002	− 0.721, − 0.234
Error rate	0.544	0.001	0.253, 0.741	0.511	0.002	0.228, 0.734
Rt. DLPFC in early period	0.394	0.019	0.082, 0.609	0.213	0.219	− 0.105, 0.474

DLPFC, dorsolateral prefrontal cortex; Rt, right; TMTA, Trail Making Test A; TMTB, Trail Making Test B

A Bonferroni correction of (0.05/24) = 0.002 was applied to account for multiple comparisons

### 1.8. Gait performance

There was a main effect of task on speed [*F* (1,33) = 11.778, P < 0.01, partial η^2^ = 0.263], stride length [*F* (1,33) = 16.644, P < 0.001, partial η^2^ = 0.335], cadence [*F* (1,33) = 47.249, P < 0.001, partial η^2^ = 0.589], and DLS [*F* (1,33) = 17.494, P < 0.001, partial η^2^ = 0.346], which demonstrated that dual-task performance resulted in slower speed, shorter stride length, lower cadence, and higher DLS than single-task walking. Additionally, the interaction effect of task × group was found on speed [*F* (1,33) = 5.339, P < 0.05, partial η^2^ = 0.139] and cadence [*F* (1,33) = 7.530, P < 0.05, partial η^2^ = 0.186], i.e., older adults had slower speed and lower cadence during the dual task compared to younger adults (Table 2).

### 1.9. PFC subregions

**Fig. 2:**
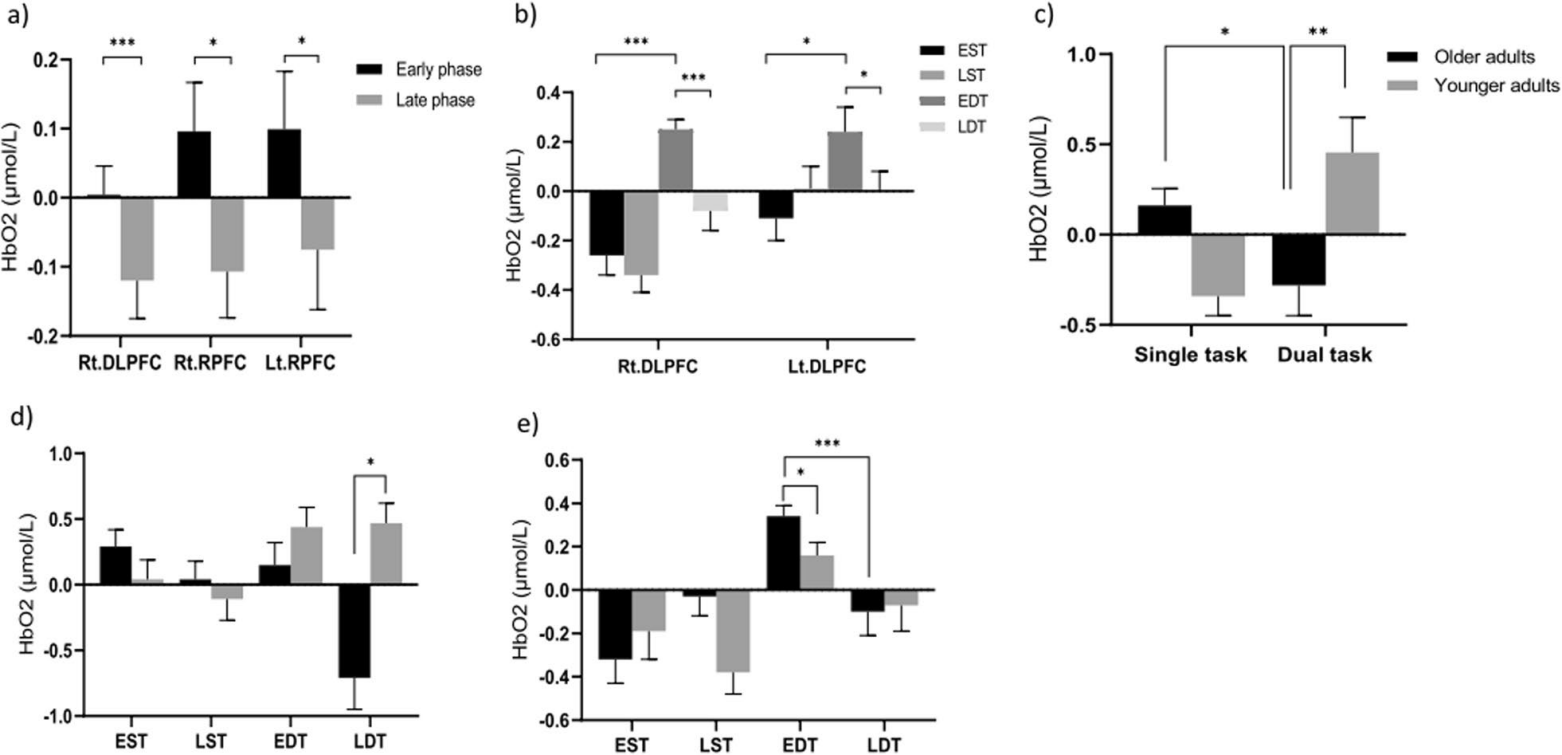
Comparison of the PFC subregions between groups. Values are presented as mean ± standard error of the mean. **a** Activity of PFC subregions in different phases, **b** Activity of PFC subregions in different phases in different tasks, **c** Rt. orbitofrontal cortex, **d** Rt. orbitofrontal cortex, **e** Rt. dorsolateral PFC. *P < 0.05; **P < 0.01; ***P < 0.001. EST, early single-task condition; EDT, early dual-task condition; LST, late single-task condition; LDT, late dual-task condition; HbO_2_, oxygenated hemoglobin; PFC, prefrontal cortex; DLPFC, dorsolateral PFC; RPFC, rostral PFC; VLPFC, ventrolateral PFC; Rt, right; Lt, left

The main effect of task was observed in the right DLPFC [*F* (1,33) = 18.596, P < 0.001, partial η^2^ = 0.360], left RPFC [*F* (1,33) = 12.399, P < 0.01, partial η^2^ = 0.273], right VLPFC [*F* (1,33) = 7.643, P < 0.01, partial η^2^ = 0.188], and left VLPFC [*F* (1,33) = 12.541, P < 0.01, partial η^2^ = 0.275], i.e., the activity of the corresponding PFC subregions during the dual task was significantly higher than that in the single task. The main effect of time was observed in the right DLPFC [*F* (1,33) = 14.480, P < 0.001, partial η^2^ = 0.305], right RPFC [*F* (1,33) = 6.396, P < 0.05, partial η^2^ = 0.162], and left RPFC [*F* (1,33) = 4.865, P < 0.05, partial η^2^ = 0.128]. This result indicated that the activity level of the corresponding PFC subregions in the early period was significantly higher than that in the late period (Fig. 2a). An interaction effect of task × time was observed in the right DLPFC [*F* (1,33) = 5.992, P < 0.05, partial η^2^ = 0.154] and left DLPFC regions [*F* (1,33) = 4.624, P < 0.05, partial η^2^ = 0.123], indicating that the activity level of the corresponding PFC subregions in the early period was significantly higher than that in the late period only in the dual task, and the activity level of the corresponding subregions in the early phase during the dual task was higher than that during the single task (Fig. 2b). Furthermore, there was an interaction effect of task × group on the right OFC [*F* (1,33) = 7.997, P < 0.01, partial η^2^ = 0.195], indicating that the activity level in the right OFC in the dual task was significantly lower than that in the single task in older adults, and the activity level in the right OFC in the dual task for older adults was significantly lower than that for younger adults (Fig. 2-c). Even though no interaction effect of task × time × group was observed, the post-hoc test showed that the activity level of the right OFC in the late period was higher in younger adults than that in older adults in the dual-task condition (Fig. 2d). There was an interaction effect of task × time × group in the right DLPFC region [*F* (1,33) = 4.307, P < 0.05, partial η^2^ = 0.115]. Post-hoc tests revealed that the activity level of the right DLPFC in the early period during the dual task was significantly higher than that in the late period for older adults, and the activity level of the right DLPFC in the early period during the dual task for older adults was significantly higher than that for younger adults, but not in the late period (Fig. 2e).

### Correlation between activation of PFC and dual-task performance

**Fig. 3:**
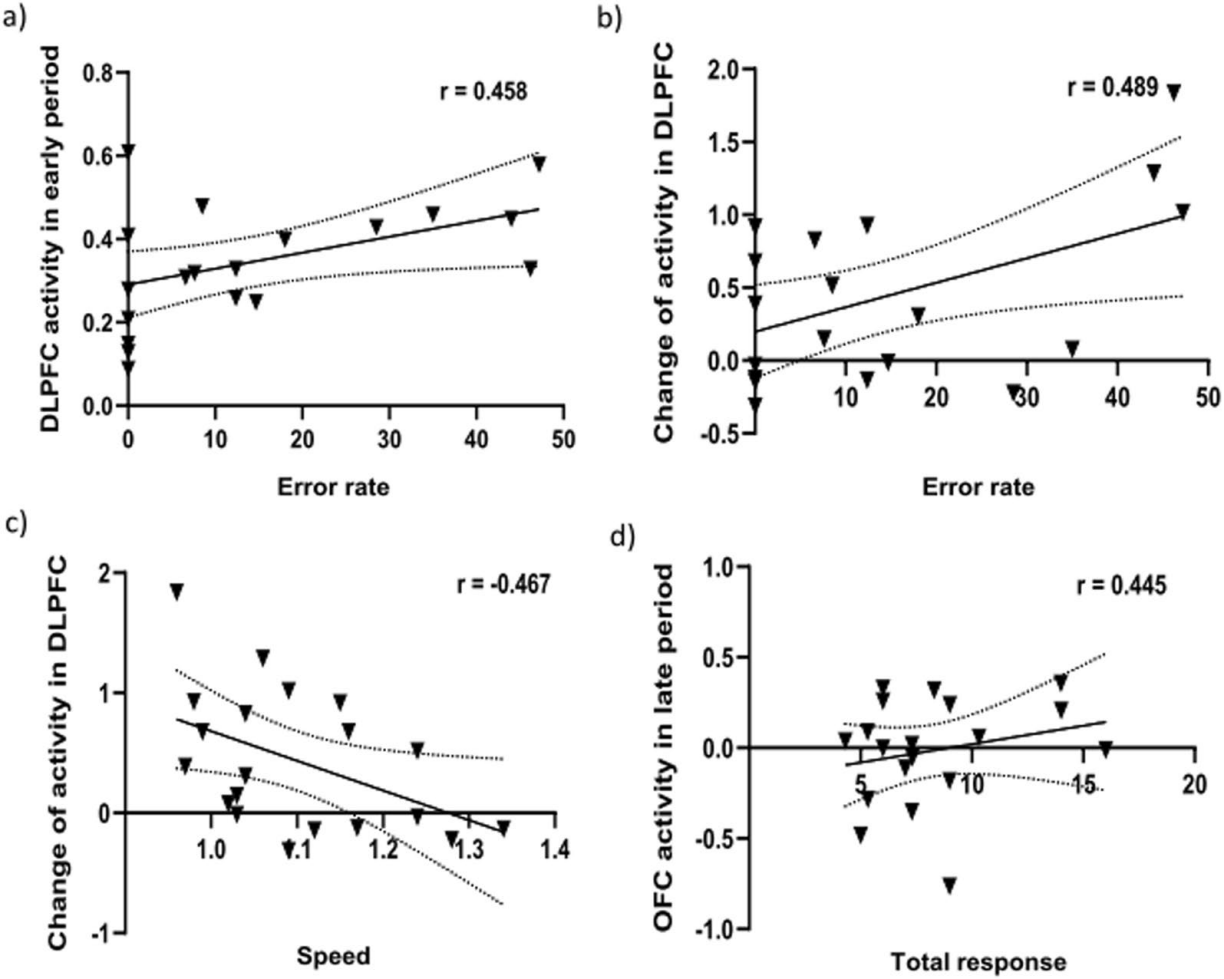
Correlation PFC subregions between dual task performance in older adults. **a** Rt. DLPFC activity in early period and error rate, **b** Change in Rt. DLPFC activity across different phases (early to late period) and error rate, **c** Change in Rt. DLPFC activity across different phases and speed, **d** Rt. OFC activity in late period and total response. DLPFC, dorsolateral PFC; HbO_2_, oxygenated hemoglobin; OFC, orbitofrontal cortex; PFC, prefrontal cortex; Rt, right

Analysis of the correlation between PFC subregions and dual-task performance in older adults revealed a significant positive association between the activity of the right DLPFC in the early period and the error rate in the dual-task condition (r = 0.458; P < 0.05; 95% CI 0.066–0.814), and a positive association between change in the activity of the right DLPFC across different phases (early-late period) and the error rate in the dual-task condition (r = 0.489; P < 0.05; 95% CI, − 0.179 to 0.789). A negative association was observed between change in the activity of the right DLPFC across different phases and speed in the dual-task condition (r = -0.467; P < 0.05; 95% CI, − 0.690 to − 0.134). Additionally, a positive association was observed between the activity of the right OFC in the late period and the total response (r = 0.445; P < 0.05; 95% CI 0.137–0.677) in the dual-task condition (Fig. 3). No significant correlation was found in younger adults.

## Discussion

The present study verified functional activity patterns in subregions of the PFC in different phases and task performance in older and younger adults during dual-task walking using fNIRS and a gait analyzer. This study focused on the phase of brain activity (early and late periods), which could help elucidate the differences in functional PFC activity patterns between younger and older adults during a dual-task with greater specificity. Furthermore, instead of measuring only a single localized region using a limited number of channels, we employed multiple channels to measure several subregions. This approach allowed us to examine the involvement of various PFC subregions in dual-task performance. The inferences of our study are as follows. First, the activity of the PFC subregions during the dual task is higher than that during the single task. Second, over-activation of the DLPFC occurred in the early period in older adults, which declined sharply in the late period, compared to younger adults. Third, the activity in the right OFC in the dual task was lower in older adults than that for younger adults. Therefore, older and younger adults have different PFC activity patterns during dual-task performance. These altered PFC subregion-specific activation patterns in older adults indicate insufficient adaptation processes for a given task and a decline in dual-task performance with age.

### 1.11. Cognitive and gait performance

Both groups showed a deterioration in performance in the dual-task condition than that in the single-task condition. Older adults showed decreased speed, stride, and cadence and increased DLS and a decline in all cognitive task variables during the dual task compared to the single task. Comparison between groups with significant interaction effects revealed that older adults showed worse performance in both domains, i.e., gait and cognition (speed, cadence, total response, correct response, accuracy rate, and error rate) during the dual task compared to younger adults. This suggests that the dual-task capacity to simultaneously accommodate and process the demands arising from the two tasks decrease because of aging, leading to deterioration in the performance of one or both tasks, which is termed dual-task interference [46, 47]. Moreover, mutual interference indicating severe deterioration in both the primary and secondary tasks, rather than mild interference in which only one task performance decreases during the dual task, was pronounced in older adults [48, 49]. In our study, mutual interference was clearly observed in older adults. Brustio et al. [49] demonstrated higher mutual interference in motor and cognitive tasks in older adults than that in younger adults while performing subtraction exercises during mobility tasks. Furthermore, the results of the TMTA and TMTB-A indicate that older adults had poorer executive function compared to younger adults, which would support the notion that the older adults in this study experienced severe mutual interference. The results also showed that the poorer the execution function, the poorer the dual-task performance (Table 3). A previous study reported that older adults with low executive function had higher PFC activity and higher dual-task interference compared to younger adults [17].

### Activity of the PFC subregions

**Fig. 4:**
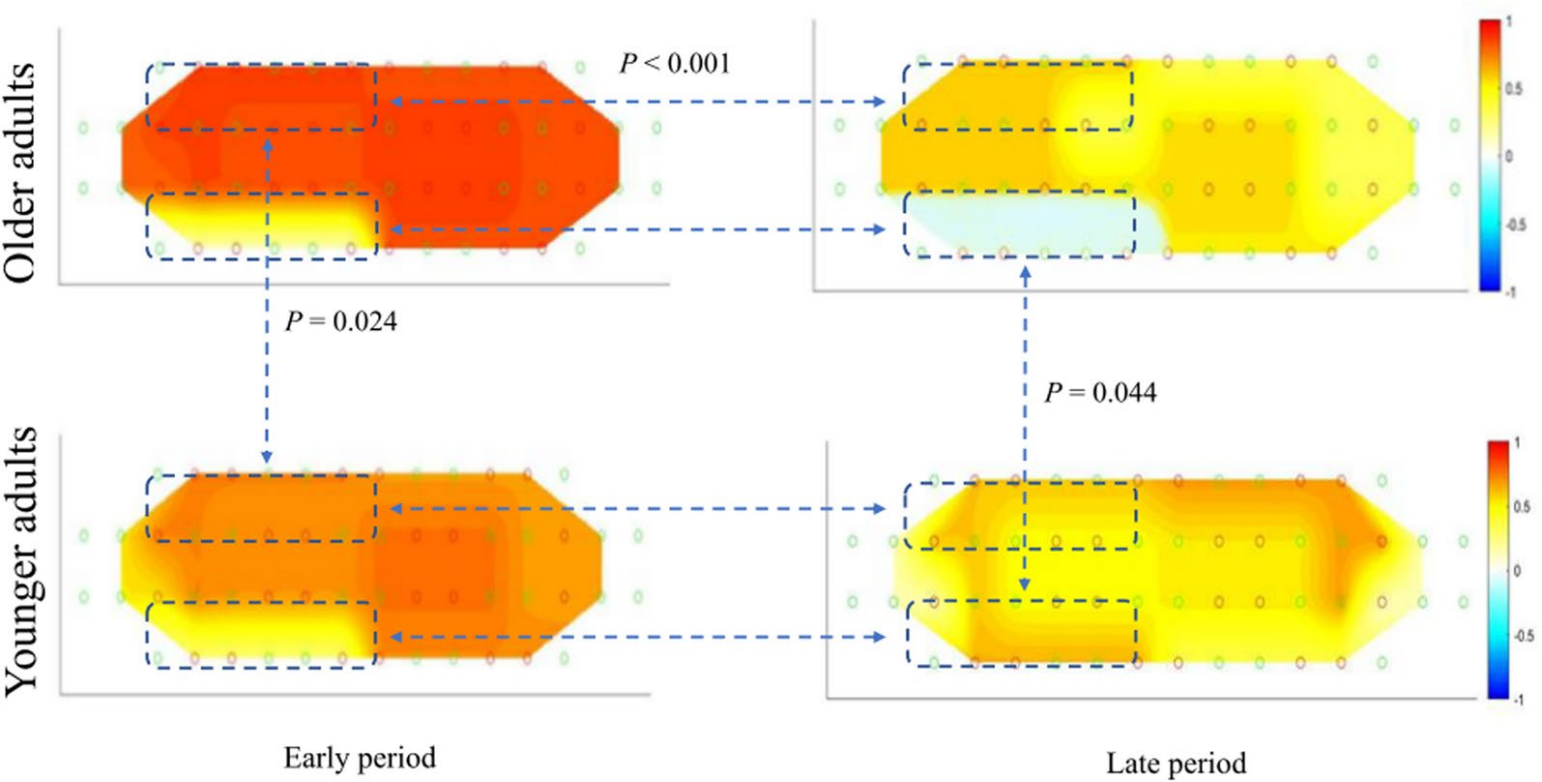
Difference of change of connectivity node in the prefrontal cortex in different phases during dual-task walking between older and younger adults

Dual-task walking led to higher activation in the PFC subregions, including the right DLPFC, left RPFC, and bilateral VLPFC compared to single-task walking, consistent with the results of previous studies [5, 9, 50]. As the DLPFC is responsible for higher-order cognitive processes, including attention shifting, working memory, and inhibition, it plays an essential role in performing both tasks simultaneously [6, 21]. The RPFC is associated with working memory and prospective memory needed to execute an intended action in the future, exerting a considerable impact on multitasking [6, 51]. The VLPFC is involved in the attention-switching function, which is required to perform dual tasks [19]. Hoang et al. reported that walking while serially subtracting sevens led to higher activity in the DLPFC than walking alone [48]. Mirelman et al. reported that dual-task walking (walking + serial subtraction of sevens) induced higher activity in the RPFC compared to single-task walking [13]. Another study reported that walking while performing a cognitive task led to higher activity in the VLPFC in younger adults [15]. Furthermore, our results showed that increased bilateral activity in the DLPFC in the early period during the dual-task plummeted in the late period. DLPFC activity underwent attenuation with repetition of the task, indicating adaptation and automaticity of the task [52, 53]. The level of PFC resource utilization for the dual task would reduce with the familiarity of the task [54]. A previous study demonstrated that the rise in PFC activity during mobility tasks reduced with repeated trials [54]. Between-group comparison revealed that older adults showed greater activity in the DLPFC in the early period during the dual task compared to younger adults, after which the activity of the DLPFC declined precipitously in the late period for older adults (Fig. 4). These altered PFC subregion-specific activation patterns (over-activation and precipitous drop) could be explained by compensation-related utilization in the neural circuit hypothesis, wherein older adults also need higher brain activity for tasks with submaximal demands compared to younger adults to compensate for the decline in aging-induced physical and cognitive abilities [7, 27, 33]. Furthermore, the ceiling of available brain resources is lowered with aging; thus, when the ceiling is reached, the levels of brain activation sharply decrease with degradation of task performance [7, 9, 17, 55]. Hawkins et al. reported greater PFC activity in the early period during the walking task in older adults than that in younger adults [54]. Compensation-related utilization impedes the sustained maintenance of increased brain activity from the early stages to the later stages [24, 56]. However, Holtzer et al. demonstrated that older adults maintained PFC activation during dual-task conditions, suggesting that methodological differences in assessing PFC activation between our study and theirs could be the underlying cause. In their study, Holtzer et al. specifically examined the time factor by analyzing differences across six consecutive trials, without comparing patterns between older adults and younger adults or addressing the subregions of the PFC [26]. In contrast, our study focused on the time factor within a single trial (40 s), distinguishing between early and late phases with 15-s intervals. Furthermore, unlike the study that employed a talking task as secondary task, our study utilized serial subtraction. This choice of task can have a significant impact on reaching limits of available brain resources [12, 14, 57]. Interestingly, older adults showed lower OFC activity during dual-task performance compared to younger adults. Measurement of OFC activity was used to predict the timing of adaptation to an unpredictable and challenging task environment [20, 58]. Lee et al. found that the DLPFC, VLPFC, and RPFC were activated initially when younger adults performed an unpredictable mobility task, to surmount the challenge (pre-adaptive phase), and the OFC was activated later (adaptive phase), leading to improvement in the impaired performance due to increased demand.[20]The transition to OFC activation from PFC subregions activation indicates ongoing adaptation to the task.[20, 58] However, the post-hoc test in our study demonstrated that the OFC was under-activated in older adults in the late period during the dual task relative to younger adults. This result indicated that adaptation for the dual task progressed to a lesser extent in older adults (i.e., deterioration in dual-task performance). Furthermore, the results of the correlation analysis also supported our findings, i.e., over-activation in the DLPFC in the early period, followed by a precipitous decline in the later period along with a decrease in OFC activity in the late period (altered PFC subregion-specific activation patterns), were correlated with degradation of dual-task performance in older adults (Fig. 3).

There are several limitations to this study. Firstly, despite conducting sample size calculations, it is challenging to generalize these results to both older and younger adults due to the small sample size. Additionally, although no statistically significant difference in gender ratio was found, it is important to note that the sample size and intergroup gender ratio should ideally be the same. Therefore, there are limitations in generalizing beyond the scope of gender. Second, this study focused on only the PFC subregions; other cerebral regions that influence the dual-task ability should also be investigated. Additionally, to accurately identify clear differences in activation patterns between older adults and young adults during the task, it would have been necessary to compare the changes among multiple subregions of the PFC using tools such as fNIRS and fMRI in conjunction. However, this comparison was not conducted. Thirdly, the study observed lateralized activity in specific subregions of the PFC, indicating that caution should be exercised when generalizing these findings to the overall functions of PFC subregions encompassing both hemispheres. Fourth, this study adopted subtraction with only one difficulty level (serial subtraction of sevens) as the secondary task. Various tasks (e.g., verbal fluency and digit-span tasks) with differing difficulty levels (serial subtraction of threes) should be employed as a secondary task to measure the change in activity patterns in the PFC subregions according to the task. Fifth, PFC activity was measured in both periods, but dual-task performance was measured across the overall period, which should be carefully addressed in further studies. Finally, we did not calculate deoxygenated hemoglobin levels. Furthermore, using multichannel fNIRS indeed necessitates the utilization of advanced approaches, such as neuron navigation, to accurately guide the placement of probes. Taking into account the changes occurring in multiple PFC subregions during different phases of dual-tasking can provide valuable insights into the functions of each subregion. As a result, we recommend incorporating measurements of diverse PFC subregions in different phases, while carefully considering the limitations. This novel study will help further understand the change in the activation pattern of the PFC subregions due to aging and provide information for designing dual-task related-studies for patients with neurological disorders with severe decline in dual-task performance.

## Conclusion

Our results suggest alteration in the PFC activation patterns and severe mutual interference in older adults relative to younger adults [9, 17, 59]. There was no difference in the activation patterns in the PFC subregions and task performance between the groups in the single-task condition, and differences were found only in the dual-task condition. These findings suggest the limitation of single-task evaluation for unmasking the progression of aging in older adults. The distinct activity patterns in the PFC subregions (DLPFC and OFC) during different phases in older adults (relative to younger adults) indicate the need for assessing all activities in the PFC subregions to explore the differences in activity patterns in the PFC between older and younger adults. The primary findings of our study are as follows: (1) dual-task performance increases the activity in the right DLPFC, left RPFC, and bilateral VLPFC compared to single-task performance; (2) overactivation of the right DLPFC occurred in the early period in older adults compared to that in younger adults, which declined precipitously in the late period; and (3) right OFC activity in the dual task was lower in older adults than that in younger adults. Therefore, older and younger adults show differential changes in the activity patterns of the PFC subregions in different phases during dual-task performance. These altered PFC subregion-specific activation patterns in older adults may lead to insufficient adaptation processes for a task and a decline in dual-task performance. The findings of this study will aid in understanding the relationship between the changes in activity patterns in PFC subregions with aging in older adults.

## Data Availability

Data are available from the corresponding authors upon reasonable request.

## Abbreviations

DLS: Double limb support
FES-I: Falls Efficacy Scale-International
fMRI: Functional magnetic resonance imaging
fNIRS: Functional near-infrared spectroscopy
GDS: Geriatric Depression Scale
HbO_2_: Oxygenated hemoglobin
HHb: Deoxygenated hemoglobin
K-MoCA: Korean-Montreal Cognitive Assessment
PFC: Prefrontal cortex
ROI: Region of interest
SPBB: Short Physical Performance Battery
TMTA: Trail Making Test A
TMTB: Trail Making Test B
DLPFC: Dorsolateral PFC
VLPFC: Ventrolateral PFC
RPFC: Rostral PFC
OFC: Orbitofrontal cortex
